# Animal models of post-acute COVID-19 syndrome: a call for longitudinal animal studies

**DOI:** 10.3389/fimmu.2025.1521029

**Published:** 2025-02-26

**Authors:** Jingyi Dai, Fanyi He, Qian Chen, Qiujing Li, Liping Zhao, Yingrong Du

**Affiliations:** ^1^ Department of Public Laboratory, The Third People’s Hospital of Kunming City/Infectious Disease Clinical Medical Center of Yunnan Province, Kunming, Yunnan, China; ^2^ International Research Fellow, Prince of Songkla University, Hat Yai, Songkhla, Thailand

**Keywords:** post-acute COVID-19 syndrome, animal models, long-term perturbations post coronavirus infection, immune tolerance, organ manifestations

## Abstract

Animal models are indispensable for unraveling the mechanisms underlying post-acute sequelae of COVID-19 (PASC). This review evaluates recent research on PASC-related perturbations in animal models, drawing comparisons with clinical findings. Despite the limited number of studies on post-COVID conditions, particularly those extending beyond three months, these studies provide valuable insights. Three hallmark features of PASC—lung fibrosis, hyperglycemia, and neurological sequelae—have been successfully replicated in animal models, paving the way for mechanistic discoveries and future medical interventions. Although most studies have reported post-COVID conditions within 14–60 days post-infection, they still offer critical reference for future long-term research. This review also explores potential mechanisms of persisting immune misfiring, a key factor in the chronicity of PASC symptoms. Moreover, challenges in modeling PASC are also discussed, including the limited genetic diversity in inbred animal strains and difficulties in accurately identifying PASC-affected individuals. To address these issues, we propose methodological improvements, such as comparing individual animal parameters with control averages and incorporating genetically diverse populations like collaborative cross models. These strategies will enhance the identification and characterization of PASC endotypes in animal studies. By integrating findings from animal models with clinical manifestations of PASC, future research can provide more valuable insights into its mechanisms and support the development of effective therapeutic strategies. Finally, we emphasize the urgent need for longitudinal studies in animal models to fully uncover the mechanisms driving PASC and guide interventions to mitigate its public health impact.

## 1 Introduction

Microbial infections can induce long-term consequences beyond acute diseases or chronic infections. In particular, repeated viral exposure plays a key role in neurodegenerative diseases (1–3). COVID-19 patients not only suffered from acute- phase disease, but also experienced highly heterogeneous post-COVID conditions (4). Furthermore, SARS-CoV-2 infection has been associated with an increased risk of developing other non-infectious diseases such as diabetes (5), cardiovascular problems (6), autoimmune diseases (7), and neurological or psychiatric disorders (8). These findings reinforce this correlation. In the post-COVID era, PASC triggered by repeated infections from new variants of SARS-CoV-2, will continue to pose a significant threat to public health. Therefore, understanding the mechanisms behind long COVID and advancing therapeutic innovations remain crucial. However, currently, several limitations hinder the identification of a clear, reproducible, and generalizable long COVID/PASC signature, including sample accessibility, disease heterogeneity, inconsistent PASC definitions, variations in sample collection timing and methodologies, and uncontrollable factors such as infection severity, reinfection, co-infection, and subsequent infections. Therefore, the development and careful characterization of relevant animal models, which can can offer better control over certain factors, are crucial for revealing the underlying mechanisms of PASC.

Animal models are powerful tools for elucidating disease pathomechanisms. While post-infection perturbations and dyshomeostasis have been widely reported in COVID-19 models (9), research specifically focused on PASC is far less extensive. A key challenge is identifying PASC in animal models and appropriate control groups, often limiting comparisons to infected versus uninfected, vaccinated, or influenza-infected animals (10, 11). Ideally, more rigorous comparisons should be made between infected animals with and without PASC (12). Furthermore, the limited intraspecific diversity of most inbred animal strains may prevent them from fully exhibiting the heterogeneous symptoms observed in humans. Although animal models may not fully recapitulate the clinical manifestations of PASC, animal models offer several advantages for PASC studies, as long as findings from animal models are reproducible. 1. Systematic analysis can be conducted in animal models at serial time points, such as systematic histopathology analysis, multiple omics analysis, and viral detection. 2. Consistent pathogenic features in animal models can be profiled and reevaluated for further research. 3. Engineered animal models or experimental interventions can be applied for PASC research. Here, we systematically reviewed recently published research (including preprints) on PASC or coronavirus infection induced long-term perturbations, and present the similarities and differences by comparing these models with clinical findings (**Table 1**). Most importantly, these studies offer a valuable reference for future PASC research. Applying improved methodologies for identifying PASC in animals will offer more valuable insights though rigorous comparisons between infected animals with and without PASC.

**Table 1:** Organ manifestations.

Time scale	Manifestations	Tendency at study endpoint	Reference
Nervous system			
31 days	At 31 dpi, permanent injury to the lung and kidney was observed, characterized by peribronchiolar metaplasia and tubular atrophy, respectively. Transcriptional alterations in lungs, kidneys, heart and brain persisted up to 31 dpi. SARS-CoV-2 infection resulted in transient olfactory dysfunction in mice, resolving by 15 dpi. Similar to clinical findings, anosmia is normally resolved within 2-3 weeks (63). Notably, SARS-CoV-2 infection in mice induced persistent olfactory inflammation and reduced burying activity at 26 dpi, an assay commonly used to assess rodent repetitive and anxiety-like behaviors	Gene set enrichment analysis revealed an expansion of pathways involved in microtubular motor activity and axoneme assembly in the lungs, consistent with bronchiolization. Prolonged elevation of canonical interferon-stimulated genes and Cxcl10 was observed in the olfactory bulb and epithelium, with a gradual decline. However, Ccl5 expression kept increasing, as well as Iba1 levels, a biomarker for microglial and macrophage activation.	(11)
28 days	Neurological changes, such as reactive astrocytes and microglia, brain hypoxia, perivascular cuffs, degeneration of Purkinje cells, brain microhemorrhages with/without vascular injury, neuronal injury and apoptosis, were found in SARS-CoV-2 infected non-human primates, only with sparse viral infection in brain. Hypoxic-ischemic injury, reactive astrocytes and microglia observed align with autopsy findings in human brains (64).	While this study did not specifically compare early and late stages, neuronal apoptosis appeared to increase over time. Apoptosis was completely absent in primates that succumbed at acute phase, while exhibited more severe microhemorrhages.	(32)
14 days	Fibrotic scarring in lung and microglial activation and perivascular blood cuffing were also observed in brain at 14 dpi, even though no viral RNA and N protein were detected at this time point. SARS-CoV-2 infection through intranasal droplets leads to lethal outcomes and is not suitable for long-COVID model.	Lung pathology worsened, and brain Iba1 levels increased over time. Permanent damage caused during the acute phase may leave long lasting consequences.	(35)
21 days	Distinct infectivity and immune patterns were observed among variants. The expression of several cytokines remained elevated in the lungs, brain, and heart up to 21 dpi, varying depending on the viral strain. A resurgence in the numbers of B cells, dendritic cells, and macrophages at 14 dpi in bronchoalveolar lavage fluid was noted. A gradual accumulation of tau pathology was observed in cortex, hippocampus, striatum, and amygdala up to 21 dpi.	The expression of several cytokines (viral strain specific) and Tua pathology (AT8 staining) increased with time.	(34)
31 days	A type 1 IFN triggered neuropathic transcriptome alteration in dorsal root ganglia, coincided with SARS-CoV-2–specific mechanical hypersensitivity mechanical hypersensitivity, was found up to 31 dpi.	An expansion of differentially expressed genes was observed at 31 dpi compared to 4 dpi	(65)
4 months	Extensive longitudinal behavioral studies in mice surviving severe SARS-CoV-2 infection revealed a broad spectrum of neurological abnormalities in neuropsychiatric state, motor behavior, autonomic function, and reflex and sensory function. Minimal astrocyte activation and minimal to mild microglial activation were detected in brain, alongside with brain transcriptomic alteration in pathway related to complement activation, phagocytosis recognition, and humoral immune response mediated by circulating immunoglobulin up to 4 mpi. Persistent complement activation is revealed as a key feature of human long-COVID (30).	Abnormalities in spontaneous activity, tail position, and tremor increased, while gait, whisker response, ear twitch, and palpebral reflex decreased over the 4-month period. Mild inflammation persisted in the brain. The levels of IL-6, CCL5, CXCL10, and CCL11 in lungs remain elevated.	(16)
61 days	Persistent mild brain lesions with gliosis and hyperemic blood vessels and neuropsychiatric sequelae were observed up to 60 days after MHV infection. Extensive behavioral examination revealed female were more susceptible for post coronavirus infection syndrome, mirroring the sex difference observed in long-COVID (67). Persistent microglial activation and increased IL-6 levels were detected in both sexes. Female exhibited reduced marble burying activity at 34 dpi and significant cognitive dysfunctions at 60 dpi. The olfactory dysfunction in female was resolved by 38 dpi.	A resurgence of increased Iba1 levels was observed in the brain at 60 dpi. IL-6 levels remained elevated, particularly in males. Elevated levels of S100B^+^ cells were detected in females	(19)
30 days	SARS-CoV-2 infection triggered monocyte infiltration and microglial activation in central nervous system. Myeloid cell-derived IL-1β impaired hippocampal neurogenesis, contributing to subsequent cognitive deficits. Adenoviral-vectored spike vaccination mitigated hippocampal degeneration.	T cells and myeloid cells remained elevated. Reduced double cortin-positive neuroblasts returned to bassline. Proliferating neuroblasts and synapses remain lower.	(10)
60 days	SARS-CoV-2 infection increases the Parkinson’s disease susceptibility and cellular toxicity in a humanized Parkinson’s disease model pretreated with human preformed fibrils by inducing a persisting astrocyte and microglial activation, even when the virus is undetectable.	Astrocyte and microglial activation were gradually subsiding.	(68)
30 days	Expanded CD8^+^ T cells found in the brain of aged mice after MHV infection caused neuronal cell death, neuronal regeneration in hippocampus, and subsequent spatial learning impairment, rather than microglial activation. These cytotoxic T cells induce neuronal apoptosis via IFNγ pathway, instead of antigen-specific killing. *in vitro*	Sustained microglial activation and elevated levels of IFN-γ and TNF-α were found in the central nervous systems of aged mice.	(18)
30/60 days	Microglial activation and lose of neuronal marker NeuN persisted in hippocampus of MA10 infection mice by 60 dpi. Perivascular lymphocyte cuffing was observed by 30 dpi.	Perivascular lymphocyte cuffing increased over time by 30 dpi.	(66, 69)
No infection	IgG from long COVID-19 patients can cause persistent sensory hypersensitivity or reduced locomotor activity in mice. These IgG can target on murine heart, skeletal muscles and spinal cord neurons.		(53)
No infection	Spike protein induced a long-term cognitive dysfunction via TLR4 signaling. Knockout of TLR4 and TLR4 blocking improves the synapse elimination and memory dysfunction.		(70)
Lung			
121 days	Aged hamsters suffered from persistent sub-pleural and interstitial pulmonary fibrosis, as well as alveolar bronchiolization, until 112 dpi after a mild physical exercise. A decrease of CK8^+^ alveolar differentiation intermediate cells and a dominated CK14^+^ airway basal cells population is correlated with bronchiolization.	Azan^+^ area, an indicator of fibrosis gradually increased by 121 dpi.	(14)
30 days	Sustained lung inflammation, injury and airway wall thickening alongside with long‐term neutrophil recruitment, fibrotic changes, and increased NET formation were observed in the lungs of mice for up to 30 dpi.	Net formation gradually increased from 5 dpi to 30 dpi.	(71)
35 days	IFNγ secreted by resident T cells recruited profibrotic macrophages to caused fibrosis in both human and mice model. Blocking IFNγ reduced lung inflammation and fibrosis.	Lung pathology and T cells peaked at 21 dpi and subsequently decline, while remained elevated by 35 dpi. IFNγ signaling waned by 35 dpi.	(12)
120 days	MA10 infection induced a heterogeneous and persistent pulmonary lesions in aged mice, mainly manifested as fibrosis and chronical inflammation in lungs. Similar severity in lungs were detected at acute phase between young and aged mice, while most young mice resolve these damage by 121 dpi.	Histopathological score and recruitment of macrophages and lymphocytes persisted up to 120 dpi.	(13)
120 days	SARS-CoV-2 persistently replicated in lungs of hamster from 42 dpi to 120 dpi, alongside with alveolar consolidation, chronic inflammation, alveolar-bronchiolization and fibrotic changes in lungs. Increased proliferation and differentiation of CK14^+^ cells lead to alveolar bronchiolization	IL13, IL33, several gene involved with fibrosis remained elevated.	(15)
31 days	SARS-CoV-2 infection induced a long-term and sex-differential changes in lung proteome of hamsters with no remaining of virus.	Seven proteins remained elevated. Persistent upregulation of Muc5AC is found in other studies (13, 15).	(72)
14 days	A dysregulated alveolar regeneration is found in hamster model by 14 dpi even with no detectable viral antigen in hamster.		(73)
Heart			
24 weeks	Spike protein of SARS-CoV-2 induced a systematic suppression of mitochondrial genes and caused cardiac fibrosis and decreased ejection fraction in obese mice.		(74)
Heart			
4 weeks	Hamsters experienced a triphasic cardiac conduction system dysfunction, which peaked at 1‐3 dpi, ceased by 7dpi and recurred and persisted up to 28 dpi. Persistent cardiac conduction system injury and dysfunction along with increased cardiac cytokines, interferon‐stimulated gene expression, and macrophage remodeling in SARS-CoV-2 infected hamster model.	Cardiac conduction system dysfunction redeveloped at 28 dpi.	(75)
Skeleton muscle			
60 days	SARS-CoV-2 infection left a long-lasting skeleton muscle atrophy and metabolisms suppression. Mitochondrial function is impaired by IFN-γ and TNF-α.	A significant decrease in myofiber cross-sectional area and an expansion of differentially expressed genes in muscles were detected by 60 dpi.	(24)
Intestine			
12 months	Nests of erythrocytosis, diffused inflammation and the infiltration of lymphocytes are found in mouse intestines 12 months after MHV-1 infection.		(21)
Testis			
4 weeks	Testicular morphological alterations, including interstitial edema, various tubular defects, and germ cell abnormalities, peaked during the acute infection phase (5 dpi). Most of these injuries resolved by 30 days post-infection, which mirrors the clinical findings (76). SARS-CoV-2 induces a transcriptomic changes in dysregulation of inflammatory, cell death, and steroidogenic pathways.	TNF and IL-6 levels in testis remained higher at 30 dpi, alongside with increased immune cells infiltration.	(23)
Gut-brain axis			
No infection	Mice that receives fecal transplantation from post-COVID patients exhibits poor cognitive performance.		(77)
Blood and liver			
18 weeks	SARS-CoV-2 infection induces a long-term elevation of blood glucose and a dysregulated blood chemokine signature in African green monkeys. No viral replication and inflammation are found in in the liver and pancreas, and an enhanced activity of gluconeogenic enzyme phosphoenolpyruvate carboxykinase is thought to cause hyperglycemia.		(17)
Skin			
12 months	The loss of hair follicles, damage to adipose tissues, and injury to epidermal layer are found in MHV infected mouse 12 months post infection.		(20)

## 2 Short and long term post-acute COVID-19 impact found in animal models

Due to the very few animal studies revealed that long-term sequelae of COVID-19 beyond three months, we employed a less stringent criteria for selecting studies investigating post-COVID conditions (9). According to the current consensus definition of PASC, only five animal studies qualify as PASC research. Three of these studies reported the fibrotic changes in rodent lungs (13–15), one reported a neurological sequelae in mice (16), and another reported hyperglycemia in a non-human primate model (17). Apart from the post-COVID studies, four studies revealed sequelae post mouse hepatic virus (MHV) infection were also included, including two studies on central nerve system impacts (18, 19) and two studies reflecting MHV-induced histopathological changes lasting for one year (20, 21). The MHV model represents a natural coronavirus infection process in mice. Although it may trigger different immune response compared with SARS-CoV-2, it may help to reveal some shared mechanisms of viral infection induced neurodegenerative diseases. Moreover, extensive longitudinal systematic histopathology examination is recommended in future PASC studies (20, 21).

**Figure 1:**
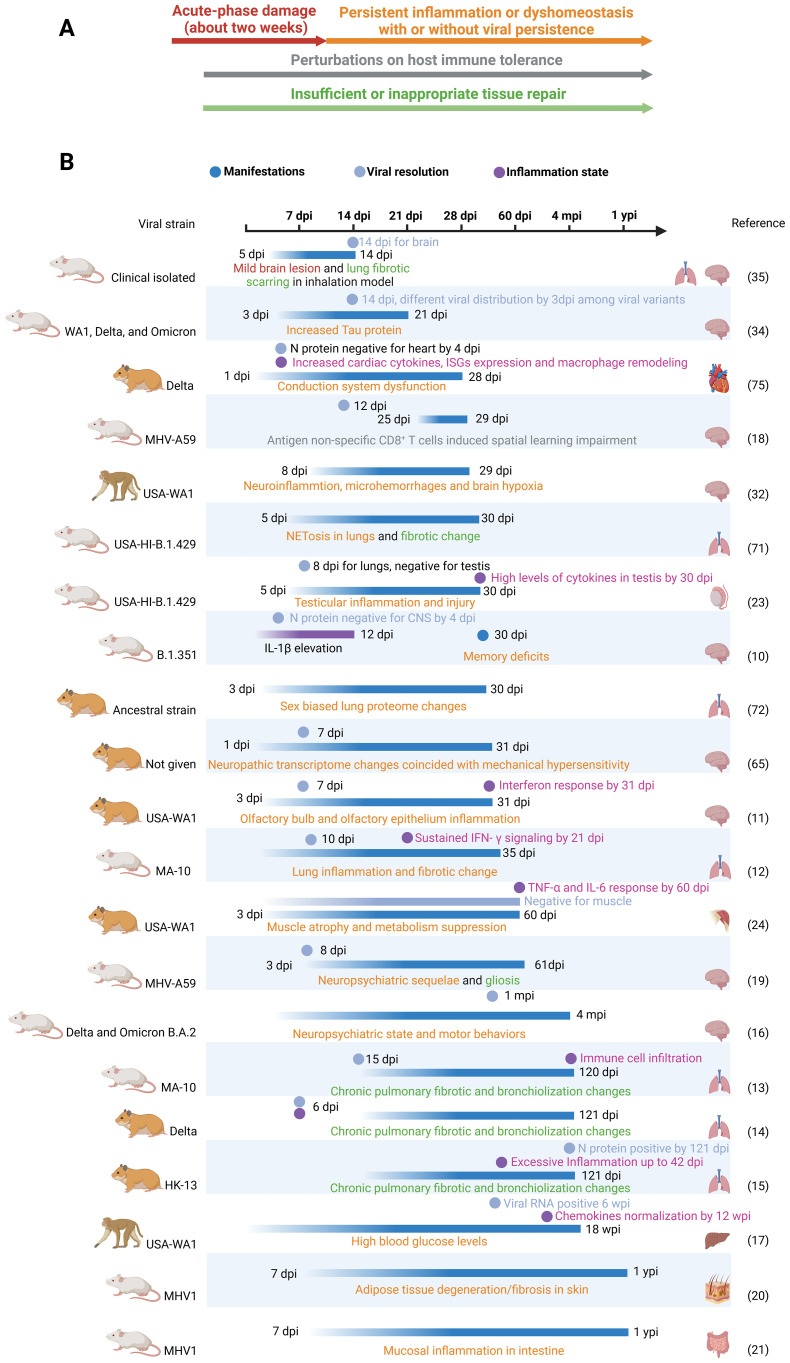
**(A)** Three pillars underlie long-COVID: acute phase damage, persistent inflammation or perturbations and maladaptive repair. Persistent inflammation can be caused by perturbations in immune tolerance (acquired immunity) and persisting activation of innate immunity by unknown reasons. In Figure **(A)**, four classes are represented by distinct colors. These colors correspond to the same classes depicted in **(B)**. **(B)** Timeframes for long-COVID animal models. In addition to only recording organ manifestations in long-COVID models, viral clearance and inflammation resolution can provide valuable insights into the underlying mechanisms. We put only one study with a duration of less than 2 weeks’ here to address the importance of acute damage. Dpi, wpi, mpi and ypi represent days, weeks, months and years post infection respectively. .

While most included studies had durations of less than three months, many observed signs of exacerbation or permanent injury initiated during the acute phase of infection. These observations suggest that these sequelae may extend beyond the study durations, thus providing valuable references for future research. However, some short-term sequelae, such as olfactory dysfunction, testicular damage, and muscle atrophy (22–24), are likely to resolve within one or two regenerative cycles. Despite this tendency toward ceasing, it remains possible for individuals to experience long-term impacts, such as persistent muscle weakness or anosmia. Therefore, we compiled the main organ manifestations observed in these studies, along with their similarities to clinical findings and PASC sequelae at the study endpoints, in **Table 1**. Future research should prioritize monitoring these manifestations over extended timeframes to reveal the mechanisms underlying PASC. Findings from these animal models substantiate three pillars underlying the PASC (**Figures 1A, B**): 1. Damage caused during the acute infection phase that does not fully recover after viral resolution. 2. Subsequent and persistent damage caused by immune perturbations or other dyshomeostasis, with or without viral persistence. 3. Maladaptive tissue repair leading to conditions like interstitial pulmonary fibrosis and alveolar bronchiolization.

While most of the included post-COVID studies reported resolution of coronavirus infection within the acute phase, two PASC studies documented prolonged viral replication: one lasting 121 dpi (15) and the other persisting for 6 weeks post-infection (dpw) (17). However, recent research suggests that viral remnants, rather than actively replicating virus, may be responsible for the neurological sequelae of COVID-19 (25). Therefore, future PASC research employing small-micelle-mediated organ-efficient clearing and labeling techniques could be crucial in determining the duration of viral remnant persistence and their presence in relevant animal models.

## 3 Animal models of PASC

### 3.1 Lungs

Lung fibrosis, a consequence of maladaptive repair mechanisms following lung damage, is a key feature of PASC, which is significantly influenced by age (26). Two studies reported fibrosis and alveolar-bronchiolization in aged rodents over a three-month period, supporting this age-related predisposition (13, 14). Notably, in BALB/c mice, while similar mortality rates and subpleural opacities were observed in both young and aged animals, aged mice exhibited slower recovery and tendency toward fibrotic changes and chronic inflammation (13). Another study demonstrated that mild treadmill exercise at 21 dpi after the Delta variant infection re-induced lung dysfunction and fibrotic changes after apparent recovery (14). Moreover, long-term fibrosis can also be triggered by chronic viral infection in young hamsters (15). Notably, shared mechanisms underlying post-COVID sequelae in lungs between young and aged hamsters involve the CK14^+^ cells’ proliferation and differentiation into SCGB1A^+^ club cells, leading to fibrosis and alveolar bronchiolization (14, 15). Mechanistically, dysregulated lung regeneration has been correlated with increased Notch3 and Hes1 protein expression (15). Given that Notch4 has been shown to hinder regulatory T-cell-mediated tissue repair and induce severe inflammation in an influenza model (27), these findings suggest that Notch signaling may disrupt normal tissue repair following viral infections.

Heterogeneity in damage resolution is observed both within the same strain and between different mouse strains, such as BALB/c and C57BL/6J (13), after MA10 infection. The resolution of fibrosis in some individuals highlights host-specific differences in damage repair. Recent research has identified IFNγ as a key factor in post-COVID respiratory sequelae. By comparing single-cell RNA sequencing data from bronchoalveolar lavage fluid of convalescent donors with and without persistent respiratory complications, researchers found that IFNγ secreted by lung-resident T cells recruits pro-fibrotic monocyte-derived macrophages, contributing to fibrosis (12). Interestingly, blocking IFNγ alleviates fibrosis only in C57BL/6J mice and has no effect in BALB/c mice following MA10 infection (12). These findings emphasize the distinct underlying mechanisms driving similar long-term pathological changes in these genetically different mouse strains.

### 3.2 Metabolism perturbations

A study reveals SARS-CoV-2 infection induces a lasting hyperglycemia for 18 weeks in African green monkeys without detectable virus or inflammation in the liver and pancreas, along with a dysregulated blood chemokine signature (14). Increased liver glycogen was found in this model, and an enhanced gluconeogenesis was thought to be a main cause of hyperglycemia. This research presents a lasting stress response in glycometabolism post-infection and highlights a link between immune response and metabolism. Apart from the previously reported pancreatitis from the same task group (28), this research provides insights into and may open a new avenue for immunometabolism perturbations after infection. Moreover, an increased risk of Type 2 diabetes is also found in children after COVID-19 (29). Future research on how an infection can induce a disorder in glycogen metabolism as well as insulin resistance is required.

### 3.3 Neurological sequelae

A four-month longitudinal study extensively monitoring mouse behavior after severe SARS-CoV-2 infection revealed a broad spectrum of neurological abnormalities in neuropsychiatric state, motor behavior, autonomic function, and reflex and sensory function (16). Minimal astrocyte activation and mild microglial activation persisted in the brain, accompanied by transcriptomic changes related to complement activation, phagocytosis, and humoral immune response and gene expression levels associated with ataxia telangiectasia, impaired cognitive function and memory recall, and neuronal dysfunction and degeneration. Notably, lingering complement activation is also revealed in PASC patients, which is correlated with the persistence of autoantibodies and antibodies against herpesviruses (30). This convergence of findings suggests a potential link between complement activation and neuropathological changes in both animal models and human PASC. Therefore, future research should focus on identifying the specific antibodies or other pathways triggering complement activation and mediating these neuropathological changes. The autoimmunity resulting from SARS-CoV-2 infection and its impact on immune tolerance will be discussed in the “Immune Tolerance” section.

Interestingly, although mild brain inflammation persisted throughout the study, some abnormalities in gait, whisker response, ear twitch, and palpebral reflex abnormalities, decreased over time. Others, including body position, grip strength, touch escape, and reach touch, remained relatively constant. Conversely, abnormalities in spontaneous activity, tail position, and tremor increased over the four-month period. These results suggest that the impact on the brain may stem from three distinct types of damage: permanent damage incurred during the acute phase, reversible damage incurred during the acute phase, and subsequent damage that gradually develops after the acute phase. The latter on aligns with the gradual exacerbation of cognitive deficits in some asymptomatic patients (31) highlighting the need for future PASC models induced by less severe infections.

## 4 Manifestations of short-term neuropathology changes

### 4.1 Persistent neuroinflammation

The most consistent finding across these studies is the persistence of neuroinflammation, primarily characterized by microglial activation (10, 11, 32), elevated levels of Iba1, elevated pro-inflammatory cytokines, such as IL-1β (10), IL-6 (16, 19), TNF-α (18), IFN-γ (18), and chemokines such as CCL5 and CXCL10 (11). While microglial activation is a normal response to infection or injury, its persistence can have detrimental effects on neuronal function and contribute to neurodegeneration. The prolonged elevation of these inflammatory mediators can disrupt neuronal signaling, impair synaptic plasticity, and promote neuronal damage or death. Notably, a research revealed gliosis in the mouse brain after MHV infection (19). Gliosis, as an end stage of microglial activation, is thought to be a main feature and driver for persistent depressive and cognitive symptoms in patients (33). In some cases, astrocyte activation (16, 32), tau pathology (34), and perivascular lymphocyte/blood cuffing (32, 35) can be salient in some animal models.

### 4.2 Neuronal damage and dysfunction

Several studies provide evidence of neuronal damage and dysfunction (32), including neuronal cell death (18, 32), impaired neurogenesis (36), and reduced synapses (10). These findings indicate that coronavirus infection can have a direct and lasting impact on neuronal function. Notably, one study revealed the neuronal apoptosis gradually increase over time in primates, and is absent in the acute phase (32), suggesting different damage in separate phases.

### 4.3 Age and sex bias changes

Aged animals often exhibit more severe and prolonged effects due to a heighten tone of basal inflammation (18), and female-specific neuropathological changes (19), which both mirroring the clinical findings. These findings highlight the importance of considering individual variability when studying the neurological impact of coronavirus infection.

## 5 Lasting immune misfiring caused by dysregulated immune tolerance

Perturbation of immune tolerance can lead to an autoimmune response. Immune tolerance serves as a supervisor in determining what our immune response should react to and what it should not react to. Perturbations of immune tolerance encompass two key concepts: failure to respond to a substance that should elicit an immune response, and responding to a substance that should be be indifferent to. During the pathogen infection, immune tolerance is expected to protect our normal tissue from immune misfiring and not to favor pathogen replication by tolerating pathogen. However, in the SARS-CoV-2 infection, it seem immune tolerance turn into malfunctioning mode to cause damage to innocent tissue, and lead to viral persistence. The process of PASC is a long-term immune misfiring of both innate and adaptive immune response triggered by infection.

### 5.1 Aspect from acquired immunity

For pathogens like viruses, the best way to evade our immunity is to mimic host molecular traits. Several studies reveal that SARS-CoV-2 can trigger self-reactive antibodies and T cells due to molecular mimicry between host proteins and virus (37, 38), which can trigger a post-infection malaise, such as multisystem inflammatory syndrome in children (39). Apart from the mimicry strategies, SARS-CoV-2 infection is known to manipulate (40) or relax (41–43) our immune tolerance and to trigger an autoimmunity (44, 45), resulting in both acute symptoms and post-COVID sequelae. For instance, autoantibodies in cerebrospinal fluid against brain antigens are found in COVID-19 patients with neurological symptoms (46). Moreover, disruption of peripheral tolerance in the pancreas caused by SARS-CoV-2 infection is thought to be a key driver of type 1 diabetes (5). Thus, within the large infected population, a subset of critical COVID-19 patients may have a previous immune tolerance dysregulation, resulting in the production of autoantibodies against immunomodulatory proteins (47) and interferons (48). This dysregulation can ensue from genetic deficiency in the NF-κB pathway (49) or age induced (50, 51) tolerance loss. Individuals in this group are more susceptible to SARS-CoV-2 infection and more likely to develop a lasting self-reactive acquired immune response, leading to immune misfiring (43). If these immune misfirings cannot be eliminated by central or peripheral tolerance, they may lead to long-term conditions.

Circulating autoantibodies have been linked to PASC, and their levels can predict PASC symptoms (46, 52). Recently, a study demonstrated that transferring IgGs from stratified PASC subgroups based on Glial Fibrillary Acidic Protein and type-I interferon expression can lead to sensory hypersensitivity or reduced locomotor activity in mouse models, depending on IgG cohorts (53). Even though the mechanisms by which these two cohorts of antibodies can trigger different symptoms in mice remain elusive, this research does necessitate the stratification of PASC patients and highlights the diverse mechanisms underlying PASC. Indeed, another research reveal similar finding that passive transfer of IgG from long-COVID patients with neurocognitive and neurological symptoms can cause increased sensitivity and pain (54). They further demonstrate these antibodies target mouse sciatic nerves, spinal cord, and meninges, which can lead to loss of balance and coordination in mice. Similar findings also reported in clinical that bispecific antibodies targeted on both spike protein and neural tissue were found in patients with neurological symptoms (55). In a mouse model, SARS-CoV-2 infection can also trigger anti-platelet factor-4 antibody production, causing coagulopathy (56). Apart from the interfering self-antigens’ function, the activation of complement system is also a key driver of PASC (30). Further screening of human extracellular and secreted proteins will help to reveal what self-antigens are the antibodies from PASC patients react to (47), similar methods can be applied in animal models with PASC.

Similar to B cell tolerance, T cell dysfunction is also considered a significant driver of PASC. A recent study found that PASC patients can maintain robust SARS-CoV-2-specific T cells for two years compared to non-PASC controls (57). However, there are no report of immune misfiring caused by autoreactive cytotoxic CD8^+^ T cells. Only one research revealed children with multisystem inflammatory syndrome had both anti-SNX8 autoantibodies and cross-reactive T cells engaged both the SNX8 and the SARS-CoV-2 nucleocapsid protein epitopes (39). Future screening of the T cells function may offer a clearer vision of the mechanisms of PASC (36).

### 5.2 Aspect from innate immunity

In a MHV post infection model, activated spike protein non-specific CD8^+^ cells were increased in the brains of aged mice and correlated with neuronal death, further leading to cognitive decline (18). The authors further revealed these CD8^+^ cells can induce primary neuronal cell death *in vitro*. Therefore, we may have underestimated the dysregulated T cell in PASC (58). The correlation of IFN-γ released by activated CD8^+^ cells with PASC patients is also reported in clinical investigations (59, 60). Similarly, these CD8^+^ were also found in an EBV infection-induced Alzheimer model (61). Moreover, expanded activated CD8^+^ cells are found to be a key signature of Alzheimer’s disease (61, 62). Thus, dysregulated T cells play a key role in dementia caused by different viral exposures. Furthermore, more research reflecting how these T cell responses leave a long-term impact on health is needed. Unlike the cytotoxic T cells, their functions relied on the target that react to. These by stander T cells in PASC can be evaluated by their populations. Therefore, future studies monitoring these by stander T cells in SARS-CoV-2 infection are required.

## 6 Challenge for PASC research in animal model and future prospect

Two main obstacles hinder the study of PASC in animal models: the limited heterogeneity of PASC symptoms due to the relatively low genetic diversity of inbred animals, and the difficulty in accurately identifying PASC-affected animals. To address the first issue, using collaborative cross populations in future PASC studies could increase genetic diversity, facilitating the identification of genetic associations with PASC. However, the challenge of distinguishing PASC-affected animals remains. Simply comparing infected animals with uninfected controls or animals infected with other viruses may cover individuals with PASC and PASC phenotypes. For instance, female mice are predisposed to developing post-coronavirus syndrome. Therefore, to better discriminate PASC phenotypes, comparing individual animal parameters with the average of the control group is necessary (16). Furthermore, comparing each individual’s pre-infection and post-infection parameters is also crucial (17). By combining these approaches with reference from human post-COVID sequelae, researchers can make more robust comparisons between animals with and without PASC, leading to valuable insights into PASC mechanisms and, ultimately, promoting the development of effective therapies.

## 7 Methods

1000 articles were scraped from Google scholar after searching for animal models of long-COVID and manually screened for eligibility.
